# AR-13, a Celecoxib Derivative, Directly Kills *Francisella In Vitro* and Aids Clearance and Mouse Survival *In Vivo*

**DOI:** 10.3389/fmicb.2017.01695

**Published:** 2017-09-11

**Authors:** Ky V. Hoang, Haley E. Adcox, James R. Fitch, David M. Gordon, Heather M. Curry, Larry S. Schlesinger, Peter White, John S. Gunn

**Affiliations:** ^1^Center for Microbial Interface Biology, Department of Microbial Infection and Immunity, The Ohio State University, Columbus OH, United States; ^2^The Institute for Genomic Medicine, Nationwide Children’s Hospital, Columbus OH, United States; ^3^Department of Pediatrics, The Ohio State University College of Medicine, Columbus OH, United States

**Keywords:** AR-13, *Francisella*, antimicrobials, eﬄux pump, celecoxib

## Abstract

*Francisella tularensis* (*F. tularensis*) is the causative agent of tularemia and is classified as a Tier 1 select agent. No licensed vaccine is currently available in the United States and treatment of tularemia is confined to few antibiotics. In this study, we demonstrate that AR-13, a derivative of the cyclooxygenase-2 inhibitor celecoxib, exhibits direct *in vitro* bactericidal killing activity against *Francisella* including a type A strain of *F. tularensis* (SchuS4) and the live vaccine strain (LVS), as well as toward the intracellular proliferation of LVS in macrophages, without causing significant host cell toxicity. Identification of an AR-13-resistant isolate indicates that this compound has an intracellular target(s) and that eﬄux pumps can mediate AR-13 resistance. In the mouse model of tularemia, AR-13 treatment protected 50% of the mice from lethal LVS infection and prolonged survival time from a lethal dose of *F. tularensis* SchuS4. Combination of AR-13 with a sub-optimal dose of gentamicin protected 60% of *F. tularensis* SchuS4-infected mice from death. Taken together, these data support the translational potential of AR-13 as a lead compound for the further development of new anti-*Francisella* agents.

## Introduction

*Francisella tularensis* subspecies *tularensis* (*F*. *tularensis*) is a remarkably infectious facultative intracellular bacterium and the etiologic agent of tularemia, a zoonotic disease. Infection can be acquired via various routes including insect bites, aerosols, or contact with mucous membranes or abrasions (Thomas and Schaffner, 2010). *F. tularensis* can be divided into three major subspecies including *tularensis* (highly virulent in humans), *holarctica*, and *mediasiatica* (Staples et al., 2006; Kugeler et al., 2009). *Francisella novicida* is a separate but closely related species (Kingry and Petersen, 2014). Inhalation of less than 10 colony-forming units of subsp. *tularensis* (Type A) can result in a fatal infection (Staples et al., 2006), while subsp. *holarctica* (Type B) is somewhat less virulent and other *Francisella* subspecies/species are considered non-pathogenic to humans (Staples et al., 2006; Oyston, 2008). Tularemia is described as a re-emerging disease with recent outbreaks reported worldwide (Hestvik et al., 2015), including in the United States (Gurcan, 2014). *F. tularensis* can be easily spread via aerosol transmission, resulting in significant morbidity and mortality on a target population (Gurcan, 2014). These traits place this bacterium as a potential biological warfare agent: a Tier 1 Biological Select Agents or Toxins as determined by the United States Department of Health and Human Services (Oyston et al., 2004; Oyston, 2008).

*Francisella tularensis* is a facultative intracellular bacterium that targets macrophages and expresses several factors that aid its ability to evade immune clearance, making bacterial clearance difficult to achieve (Jones et al., 2012). While a subsp. *holarctica* live attenuated vaccine strain (LVS) has been used to protect against tularemia in government personnel and in other countries, there is no currently approved United States vaccine. Fluoroquinolones (e.g., ciprofloxacin), aminoglycosides, and tetracyclines comprise the antibiotics most frequently used to treat tularemia (Maurin, 2014). While no naturally acquired resistance to these antibiotics has been observed in isolates of *F. tularensis* (Urich and Petersen, 2008), the exposure of the bacterium *in vitro* to slowly elevated concentrations of ciprofloxacin can select for resistant strains, including bacteria exhibiting cross-resistance to other aminoglycosides and fluoroquinolones (Sutera et al., 2014). Additionally, sub-species of *F. tularensis* have differing sensitivities to clinically relevant antibiotics, complicating treatment options for patients (Origgi et al., 2014). Although antibiotic treatment is often successful, several treatment failures and relapses of symptoms have been described (Perez-Castrillon et al., 2001; Kosker et al., 2013). Furthermore, antibiotic resistant strains can be created for bioweapon purposes. These situations together highlight an urgent need for new drugs with novel mechanisms of action against *F. tularensis*. New therapeutic approaches have been recently investigated, including some of the most recently approved antibiotics (e.g., tigecycline, ketolides, fluoroquinolones) as well as improved delivery of antibiotics *in vivo* (e.g., liposome delivery) (Boisset et al., 2014). In addition, host-targeted therapy (Hoang et al., 2016), innate immune response enhancement by antimicrobial peptides, and combinatorial approaches with conventional antibiotics and immune adjuvants have been examined (Pammit et al., 2004; Boisset et al., 2014).

**FIGURE 1:**
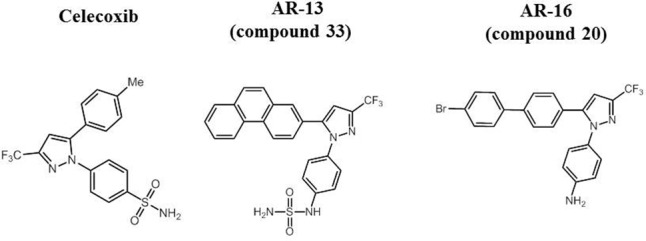
Molecular structure of celecoxib and its derivatives AR-13 and AR-16.

Previous studies showed that AR-12, a small molecule derived from the cyclooxygenase-2 (COX-2; target for some non-steroidal anti-inflammatory drugs) inhibitor celecoxib, but lacking the COX-2 inhibitory activities, displayed broad-spectrum host-directed antimicrobial activity against fungi (Baxter et al., 2011), *Salmonella enterica* serovar Typhimurium (*S.* Typhimurium) and *F. tularensis* (Chiu et al., 2009a,b; Hoang et al., 2014; Hoang et al., 2016), as bacterial burdens in host macrophages were significantly reduced in part through the induction of autophagy. Several celecoxib derivatives exhibit direct antibacterial activities against methicillin-resistant *Staphylococcus aureus* (Chiu et al., 2012), multidrug resistant tuberculosis (Salunke et al., 2015), and *Francisella* (compound 20, herein called AR-16) (Chiu et al., 2009c). In this study we report that AR-13, an AR-12 derivative with known antimicrobial activity against multi-drug resistant *Mycobacterium tuberculosis* and *Staphylococcal* spp. [compound 33 in (19)], has direct antimicrobial activities against *Francisella* species with distinct modes of action compared with AR-16. The difference between AR-13 and AR-16 lies in the substituents around the 4-[3-(trifluoromethyl)-1H-pyrazol-1-yl] aniline core. While AR-13 consists of a phenanthrene ring system at the C5 position of the heterocycle, AR-16 has a 4-bromobiphenyl group at that position. Furthermore, the aniline has been converted to a sulfamide in the case of AR-13 (see **Figure 1** for compound structures). While AR-13 displays bactericidal effects, AR-16 exhibits bacteriostatic activities against subsp. *holarctica* (strain LVS) and subsp. *tularensis* (strain SchuS4). Examination of AR-13 resistance mechanisms in the LVS strain illustrated that decreased susceptibility to AR-13 *in vitro* could be mediated by eﬄux pumps, as an eﬄux pump inhibitor sensitized the AR-13 resistant mutant to AR-13. Cytotoxicity studies revealed that AR-13 displays minimal toxicity to the human monocyte-derived macrophages (hMDMs). Finally, *in vivo* examination of AR-13 in a mouse model of tularemia showed that AR-13 treatment partially protects the LVS-infected mice from death. Combination of AR-13 with a sub-optimal dose of gentamicin provided increased protection against *F. tularensis* SchuS4 infection.

## Materials and Methods

### Bacterial Strains and Reagents

A library of celecoxib and its derivatives was kindly provided by Arno Therapeutics, Inc. (Flemington, NJ, United States). Chemical structures of celecoxib, AR-13 and AR-16 are presented in **Figure 1**. Gentamicin, kanamycin, ethidium bromine, and carbonyl cyanide *m*-chlorophenyl hydrazone (CCCP) were purchased from Sigma (St. Louis, MO, United States). *F. tularensis* SchuS4 strain (Type A), and *F. tularensis* LVS used in this study were described previously (Clay et al., 2008; Dai et al., 2012; Hoang et al., 2016). When needed, *F. novicida* mutant strains were obtained from BEI Resources transposon library^1^ (Gallagher et al., 2007). The bacteria were cultured on chocolate II agar (CHA) plates (Becton Dickinson, Sparks, MD, United States) or modified Tryptic Soy Broth (mTSB) or agar (Hoang et al., 2012) for 48 h (*F. tularensis* SchuS4 and LVS) or for 24 h (*F. novicida*) at 37°C prior to use in all experiments. Experiments involving the LVS and *F. novicida* strains were performed in a BLS2 environment. Experiments with *F. tularensis* SchuS4 were performed in The Ohio State University BSL3 select agent facility in accordance with CDC and locally approved BSL3 facility and safety standards.

### Antimicrobial Susceptibility Testing

The susceptibilities of *Francisella* to AR-13, AR-16 and other antimicrobials were determined by minimum inhibitory concentrations (MICs) and bacterial killing assays. MIC assays were performed using the microtiter broth dilution method with an initial inoculum of approximately 10^6^ bacteria/ml as described previously (Hoang et al., 2011, 2012). MICs were determined by the lowest concentration of specific antimicrobial showing complete inhibition of bacterial growth after 24 h of incubation at 37°C. For the bacterial killing assays, bacterial strains were grown at 37°C in mTSB or agar by supplementing with 135 μg/ml ferric pyrophosphate and 0.1% cysteine hydrochloride at 37°C for 48 h. Bacteria were suspended in phosphate buffered saline (PBS) to an optical density (OD) of 0.4 at 600 nm, equivalent to 3 × 10^9^ CFU/ml. Approximately 10^9^ or 10^6^ bacteria in 1 ml of PBS or mTSB, respectively, were incubated with 10 μg AR-13 or AR-16 or control dimethyl sulfoxide (DMSO) at 37°C. Viable bacteria at different time-points were evaluated by serial dilution and plating on mTSB agar plates or CHA plates.

### *In Vitro* Selection of AR-13 Resistant LVS

To examine the mechanism of AR-13 action, AR-13 was used to select for spontaneous AR-13-resistant (AR-13^r^) mutants *in vitro* by stepwise selection in broth culture. Briefly, 50 μl from an overnight culture of LVS was exposed to increasing concentrations of AR-13 in 5 ml mTSB broth with 1.25 μg AR-13/ml as a starting concentration at 37°C while shaking. 50 μl of this LVS grown culture was then passed into twofold increasing concentrations of AR-13 in 5 ml mTSB. The process was repeated until LVS was able to stably grow in 20 μg AR-13/ml (approximately 20 passes). Approximately 70 AR-13^r^ clones were selected to determine the MIC to AR-13. Two representative AR-13^r^ mutants were then passed in non-selective AR-13 free mTSB for 10 passes of an overnight culture. The stable AR-13^r^ mutants were chosen for the subsequent studies including genomic sequencing, RNA sequencing, and assays regarding the mechanism of AR-13 resistance.

### Comparative Genomic and Transcriptomic Studies

Genomic DNA from wild-type LVS and its stable isogenic AR-13 resistant mutants were purified using the GenElute Bacterial Genomic kit (Sigma–Aldrich, Cat #PLN70). Samples were sequenced on the Illumina MiSeq instrument using paired-end 2 × 300 bp chemistry, generating more than 3 million read pairs per sample. Churchill^2^ (Kelly et al., 2015) was run to identify any statistically supported variants, utilizing BWA_MEM v0.7.12^3^ and the GCF_000009245.1 assembly of the *F. tularensis* LVS reference from NCBI^4^ for alignment and GATK HaplotypeCaller v3.5^5^ for variant calling. Variants were annotated with SnpEff v4.1^6^ against the GCA_000009245.1.26 annotation database.

Similarly, the RNA from the wild-type LVS and stable isogenic AR-13 resistant mutants was purified from mid-log cultures in mTSB using the RNAeasy plus mini kit (Qiagen, Cat#74134). Using methods described previously (Jones et al., 2014), after determining the quality of total RNA using Agilent 2100 bioanalyzer and RNA Nano Chip kit (Agilent Technologies, CA), rRNA was removed from 2 μg of RNA with Ribo-Zero^TM^ rRNA removal kit for Gram-Negative bacteria (Epicentre Biotechnologies, WI). To generate directional signal in RNA seq data, libraries were constructed from first strand cDNA using ScriptSeq^TM^ v2 RNA-Seq library preparation kit (Epicentre Biotechnologies, WI). rRNA-depleted RNA (50 ng) was fragmented and reverse transcribed using random primers containing a 5′ tagging sequence, followed by 3′ end tagging with a terminal-tagging oligo to yield di-tagged, single-stranded cDNA. After magnetic-bead based purification, the di-tagged cDNA was amplified by limit-cycle PCR using primer pairs that anneal to tagging sequences have adaptor sequences required for sequencing cluster generation. The AMPure XP System (Beckman Coulter) was used to purify RNA-seq libraries. The Agilent 2200 TapeStation using High Sensitivity D1000 ScreenTape was used to determine the quality of libraries, which were then quantified using the Kappa SYBR^®^Fast qPCR kit (KAPA Biosystems, Inc., MA, United States). On average, 21 million paired-end 150 bp RNA-Seq reads were generated using the Illumina HiSeq 4000 platform for each sample (the range was 19 to 24 million). Each sample was aligned to the GCF_000009245.1 assembly of the *F. tularensis* LVS reference from NCBI^7^ using version 0.7.5a of BWAMEM^8^. The GFF file provided with the GCF_000009245.1 assembly from NCBI was used to identify transcript features and raw coverage counts were calculated using HTSeq^9^. After normalization of the RNA-Seq gene expression data a post-alignment statistical analyses was performed using custom analysis scripts written in R and DESeq2^10^. Normalized read counts were used for comparisons of gene expression and associated statistical analysis of the different bacteria/conditions. Fold change values were displayed as test condition/control condition, where values less than one were expressed as the negative of its inverse (note that there will be no fold change values between -1 and 1, and that the fold changes of “1” and “-1” represent the same value). A false discovery rate of 10% (DESeq2 adjusted *p*-value <= 0.1) was used in determining transcripts that were significantly differentially expressed.

### Isolation of Human Monocyte-Derived Macrophages (hMDMs)

Human monocyte-derived macrophages (hMDMs) were derived from human blood acquired via venipuncture from healthy donors following a Ohio State University Institutional Review Board approved protocol. Written informed consent was provided by study participants. The protocol followed published methods (Schlesinger et al., 1990; Hoang et al., 2016). Briefly, peripheral blood mononuclear cells (PBMCs) were isolated from heparinized blood over a Ficoll cushion (GE Healthcare Bio-Science, Piscataway, NJ, United States). PBMCs were then cultured in sterile screw-cap Teflon wells in RPMI 1640 plus L-glutamine (Gibco-Life Technologies, Grand Island, NY, United States) with 20% autologous human serum at 37°C in a humidified incubator containing 5% CO_2_ for 5 days. Teflon wells were chilled on ice to recover the PBMCs, which were then re-suspended in RPMI 1640 with 10% autologous serum. Cells were then allowed to attach in 24-well or 6-well tissue culture plates for 2–3 h at 37°C in a humidified incubator with 5% CO_2_. After washing to remove the lymphocytes, hMDM monolayers were seeded at a density of approximately 2.0 × 10^5^ cells/well for 24-well plates for infection studies.

### Cytotoxicity Assays

Human monocyte-derived macrophages viability was assessed in the presence of AR-13 and AR-16 using a lactate dehydrogenase (LDH) assay (Roche Applied Science, Indianapolis, IN, United States) described previously (Hoang et al., 2014). hMDM cells were seeded into 24-well plates at 2 × 10^5^ cells/well with RPMI 1640 supplemented with 20% autologous serum. This medium was replaced with 2% autologous serum in RPMI 1640 containing different concentrations of AR-13 or AR-16 (in 0.1% DMSO) or DMSO vehicle control. Triton X-100 (0.1%) was used as a positive control. After 24 h or 48 h of treatment, supernatants were collected, centrifuged to remove cells, and subjected to the LDH assay (as per manufacturer’s instructions). LDH release was measured (570 nm) and the cytotoxicity was calculated as a percentage of the Triton X-100 treated cells (positive control).

### Analysis of Bacterial Growth in hMDMs

Live vaccine strain growth in hMDMs was performed as described previously (Chiu et al., 2009b) with a minor modification. Briefly, LVS were grown at 37°C in mTSB or on agar plates supplemented with 135 μg/ml ferric pyrophosphate and 0.1% cysteine hydrochloride at 37°C for 48 h. Bacteria were equilibrated in PBS (OD_600_ of 0.4 which is approximately 3 × 10^9^ CFU/ml). LVS was added to hMDMs at a multiplicity of infection (MOI) of 50 in the presence of 2% autologous serum in RPMI 1640 plus L-glutamine (Gibco-Life Technologies). Extracellular bacteria were removed 2 h post-infection by addition of 50 μg/ml of gentamicin (Gibco-Life Technologies) for 1 h followed by three washes of the monolayer with pre-warmed RPMI 1640 to remove additional extracellular bacteria. Various concentrations of AR-13 were then added to culture medium (with 2% autologous serum and 10 μg/ml gentamicin). As a control, the parental compound of AR-13, AR-12, which was previously shown to inhibit *Francisella* growth in macrophages via induction of autophagy, was included. Soluble AR-12 and AR-13 were dissolved in 10 mg/mL in DMSO and diluted in RPMI 1640 containing 2% autologous serum to the appropriate concentrations. At 22 h post-treatment, the infected hMDMs were lysed with 0.1% Triton X-100 (Calbiochem, San Diego, CA, United States) in PBS for 15 min. The cell lysates were then serially diluted with PBS, plated onto CHA agar and enumerated after 72 h incubation at 37°C.

### Mice

Six–eight weeks old pathogen free female BALB/c mice were purchased from Harlan Sprague (Indianapolis, IN, United States). Food and water were provided *ad libitum* to the mice (5 mice/group unless otherwise indicated) in sterile micro isolator cages and allowed to acclimate for 2–3 days prior to challenge. All experimental procedures were carried out in strict accordance with guidelines established by The Ohio State University Institutional Animal Care and Use Committee (IACUC), and all efforts were made to minimize animal suffering.

### Mouse Infection with *F. tularensis* SchuS4 or LVS

Intranasal (I.N.) infection of *F. tularensis* SchuS4 and LVS was performed as previously described (Hoang et al., 2016). Briefly, *F. tularensis* SchuS4 and LVS were grown on CHA plates for 48 h at 37°C. The bacteria were removed from the agar surface and suspended in PBS to an OD_600_ of 0.4, which is approximately 3 × 10^9^ CFUs/ml. After culture dilution, mice were infected with 10 CFU of F. tularensis SchuS4 in 50 μl PBS. For LVS infection, mice were infected by the I.N. route with 3 × 10^3^ CFU in 50 μl PBS. Both doses are ∼10x the lethal number of CFU. Prior to the infection, mice were anesthetized with isoflurane as approved by The Ohio State University Institutional Animal Care and Use Committee (IACUC).

### Evaluation of Protective Efficacy of AR-13 in LVS and *F. tularensis* SchuS4-Infected BALB/c Mice

To evaluate AR-13 as a treatment for infection, survival studies were performed with *F. tularensis* SchuS4 and LVS strains. Mice were infected with *F. tularensis* SchuS4 or LVS and treated, starting about 1 h post-infection, with different doses (2.5, 5.0, or 10 mg/kg/day for LVS and 2.5 or 5.0 mg/kg/day for *F. tularensis* SchuS4) of AR-13 in 200 μl of polyethylene glycol (PEG) PEG400:0.9% saline:ethanol (50:35:15) given by the intraperitoneal (I.P.) route (Hoang et al., 2016) once daily from day 0 until day 10 post-infection. The infected mice were monitored for survival up to 2 weeks post-infection. To determine the effects of AR-13 on bacterial growth in the infected mice, we infected mice (5 mice/group) with the LVS strain via the I.N. route and treated once daily with 5 mg AR-13 in 200 μl PEG/kg/day from day 0 (the day of infection). The bacterial burdens in the lungs of surviving mice were determined by homogenization of lung tissue followed by plating and subsequent CFU enumeration.

### Statistical Analysis

Data are presented as mean ± standard deviation (SD). *P*-values were calculated using one-way ANOVA for multiple comparisons and adjusted with Bonferroni’s correction; ^∗^*p* < 0.05, ^∗∗^*p* < 0.01, ^∗∗∗^*p* < 0.001; NS, not significant. A Chi-square test was used for survival analysis. GraphPad Prism 6 was used for statistical analysis.

### Accession Numbers

RNAseq data were deposited into the GEO Repository^11^ under the record number GSE100069. Genomic sequences and alignment data have been deposited to the Sequence Read Archive^12^ under identifier SRP109660.

## Results

### *In Vitro* Susceptibilities of *Francisella* to AR-13

**FIGURE 2:**
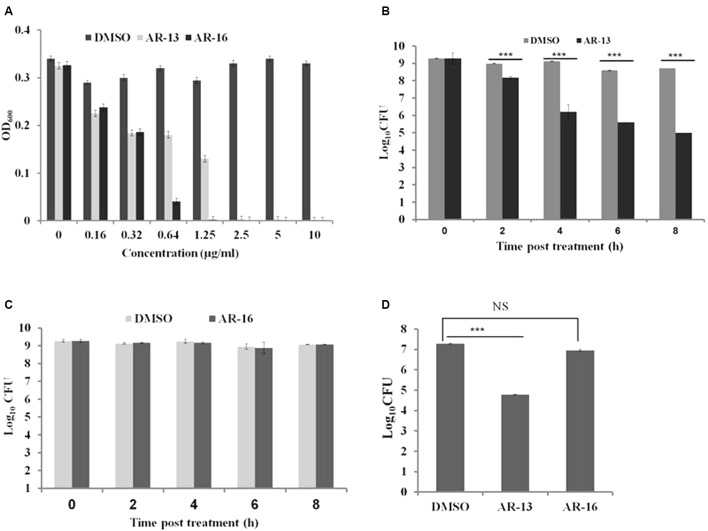
Susceptibilities of *Francisella tularensis* to AR-13 and AR-16. **(A)** LVS was grown in twofold serial dilutions of AR-13 and AR-16 in mTSB. Optical densities at 600nm (OD_600_) were measured by a plate reader 18 h after inoculation. **(B)** AR-13 has a bactericidal effect on LVS. Approximately 1.5 × 10^9^ CFU were incubated in 1 ml PBS containing 10 μg of AR-13. Viable bacteria were evaluated at different time points by serial dilution and plating. **(C)** AR-16 has a bacteriostatic effect on LVS. Approximately 1.5 × 10^9^ CFU were incubated in 1 ml PBS containing 10 μg of AR-16. Viable bacteria were evaluated at different time-points by serial dilution and plating. **(D)** AR-13 and AR-16 have bactericidal and bacteriostatic effects, respectively, on *F. tularensis* SchuS4. Approximately 1.5 × 10^9^ CFU of *F. tularensis* SchuS4 was incubated in 1 ml PBS containing 10 μg of AR-13 or AR-16. Viable bacteria were evaluated at 8 h post-treatment by serial dilution and plating. Data are representative of 2–4 independent experiments, each performed in triplicate; ^∗∗∗^*p* < 0.001; NS, not significant.

In our continuing effort to find novel antimicrobial agents to control *Francisella* infection, we screened 64 compounds derived from celecoxib (Salunke et al., 2015) to identify potential anti-*Francisella* agents using a standard serial dilution method. We found that two compounds, AR-16 and AR-13, have the ability to inhibit the growth of several *Francisella* subspecies with MICs at 24 h post-inoculation of 2.5 μg/ml for *F. tularensis* LVS (**Figure 2A**) and *F. tularensis* SchuS4 (data not shown), and 5 μg/ml for *F. novicida* (data not shown). To explore the modes of action of these two anti-*Francisella* agents, we performed bacterial killing assays the presence of 10 μg/ml for each compound in PBS (**Figure 2**) or mTSB (Supplementary Figure 1). Viable bacteria were evaluated at different time points post-treatment by serial dilution, plating and enumeration. To our surprise, the two compounds had distinct modes of action: AR-13 had bactericidal activities and AR-16 had bacteriostatic effects on LVS and *F. tularensis* SchuS4 at stationary phase (**Figures 2B–D** and Supplementary Figure 1) and at log phase (data not shown). AR-13 treatment (10 μg/ml) lead to an approximate 2–3.5 log decrease in CFUs of *F. tularensis* SchuS4 and LVS at 8 h post-treatment (**Figures 2B,D** and Supplementary Figure 1A), while AR-16 showed no significant decrease over this time period (**Figures 2C,D** and Supplementary Figure 1B). Since AR-16 was not bactericidal, we focused on our further studies on AR-13. We examined the antimicrobial activities of AR-13 on two Gram-positive bacteria, *Listeria monocytogenes* strain 10403S and methicillin-resistant *S. aureus* strain JE2 (MRSA, USA300LAC), as well as two Gram-negative bacteria, *Pseudomonas aeruginosa* strain PAO1 and *Salmonella* Typhi. The results showed that AR-13 exerted strong antimicrobial activities on the two Gram-positive bacteria, but not on the two Gram-negative bacteria (Supplementary Figure 2). These data provide strong evidence that AR-13 may serve as a potential antimicrobial with bactericidal properties to control *Francisella* and possibly other bacterial infectious agents.

### Effects of AR-13 on LVS Growth in Human Monocyte-Derived Macrophages (hMDMs)

**FIGURE 3:**
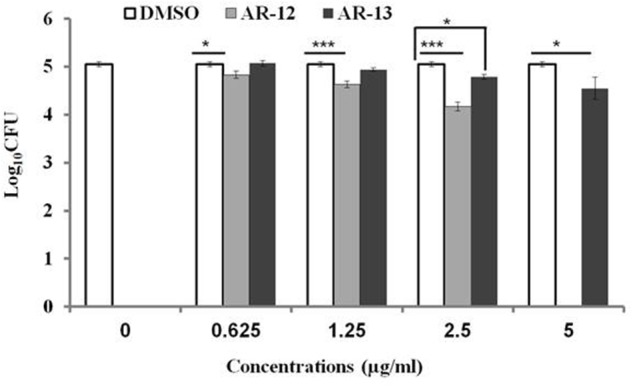
Intracellular growth of LVS in hMDMs following AR-13 treatment. hMDMs (MOI = 50) were infected for 2 h, washed and treated with different concentrations of AR-13 or its related compound AR-12, which was previously shown to inhibit *Francisella* via induction of autophagy. Intracellular bacterial CFUs were determined by plating cell lysates at 22 h post-infection. A negative control was media containing (0.05% v/v) DMSO. The experiment was repeated three times with hMDMs from three different donors with similar results and the data from a representative experiment are presented as average ± SD. ^∗^*p* ≤ 0.05, ^∗∗∗^*p* ≤ 0.001, NS, not significant.

Since AR-13 exerts bactericidal effects on *Francisella in vitro*, we sought to examine the effects of AR-13 on the growth of LVS in its primary cellular target, macrophages. The cytotoxicity of AR-13 on hMDMs was tested by measuring lactate dehydrogenase (LDH) release in the culture supernatants from hMDMs treated with various concentrations of AR-13 at 24 and 48 h post-treatment (Hoang et al., 2014). AR-13 was not toxic toward hMDMs at AR-13 concentrations as high as 20 μg/ml (Supplementary Figure 3). hMDMs were then infected with LVS and treated with different concentrations of AR-13. The intracellular bacterial load was evaluated at 22 h post-treatment. As shown in the **Figure 3**, AR-13 (at 2.5 and 5 μg/ml) significantly inhibited the growth of LVS in hMDMs, reducing the CFU recovered by ∼0.5 logs. As a control, AR-12, known to inhibit intracellular *Francisella* growth by the induction of autophagy (Chiu et al., 2009b), reduced LVS growth approximately 1 log at 2.5 μg/ml and was toxic to macrophages as expected at 5 μg/ml (Hoang et al., 2014).

### Development of AR-13 Resistance in *Francisella*

**FIGURE 4:**
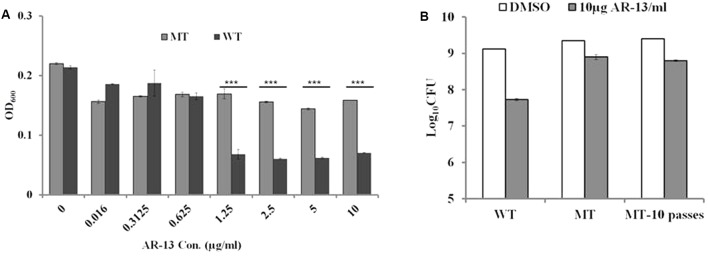
Stability of AR-13 resistance. **(A)** Growth curve of LVS (WT) and the LVS AR-13 resistant mutant (MT) in the presence of various concentrations of AR-13 in mTSB. WT or MT were grown in twofold serial dilutions of AR-13 with a starting concentration of 10 μg/ml. Optical densities at 600nm (OD_600_) were measured by a plate reader at 18 h after inoculation. **(B)** AR-13 resistance is stable. Approximately 1.5 × 10^9^ CFU of WT or MT after 10 overnight passes in AR-13-free mTSB (MT-10 passes) were incubated in 1 ml PBS containing 10 μg AR-13. Viable bacteria were evaluated at 3 h post-treatment by serial dilution and plating. Data were representative of 2–4 independent experiments each performed in triplicate; ^∗∗∗^*p* < 0.001; NS, not significant.

Examination of the resistance of microbes to new antimicrobials provides insight into the mechanism(s) of action of the drug as well as drug targets. We sought to examine whether *Francisella* was able to develop resistance to AR-13 *in vitro*. Our first attempt to select AR-13 resistant mutants by plating LVS on mTSB agar plates containing AR-13 at 4x MIC (10 μg/ml), 8x MIC (20 μg/ml), and 16x MIC (40 μg/ml) was unsuccessful. Stepwise selection in mTSB broth with increasing concentrations of AR-13 was performed to attempt to select for AR-13 resistant mutants of LVS. After approximately 20 overnight passages in increasing AR-13 selective pressure, AR-13 resistant mutants were obtained and sensitivity to AR-13 was examined by culturing in mTSB with serial concentrations of AR-13 (0.016–10 μg/ml). As shown in **Figure 4A**, a representative AR-13 resistant mutant was able to grow in the presence of up to 10 μg/ml AR-13 but was inhibited in 20 μg/ml (eightfold increase in MIC) (data not shown). Examination of the stability of an AR-13 resistant mutant was performed by passaging the mutant in AR-13-free mTSB for 10 overnight passages, with several clones then chosen for bacterial killing assays. As shown in **Figure 4B**, after 10 overnight passages in AR-13-free mTSB, there was no difference in viable bacteria recovered between the initial AR-13 resistant mutant (MT) and that of 10 overnight passages in AR-13-free mTSB (MT-10 passages). These data suggest that *Francisella* is able to develop resistance to AR-13 and the resistance is relatively stable.

### Mechanisms of AR-13 Resistance in *Francisella*

**FIGURE 5:**
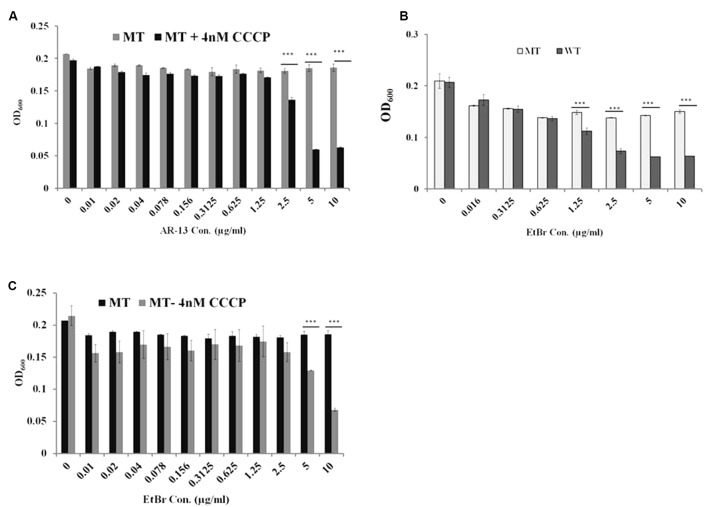
Eﬄux pumps mediate AR-13 resistance in LVS. Eﬄux pump inhibitor CCCP sensitizes the AR-13 resistant (AR-13^r^; MT) mutant to AR-13. **(A)** The MT was grown in twofold serial dilutions of AR-13 with or without a sub-inhibitory concentration of CCCP (4 nM, see Supplementary Figure 4 for more information). OD_600_ values were obtained by a plate reader at 18 h post-inoculation. **(B)** AR-13 resistance confers EtBr resistance in LVS. WT and MT were grown in twofold serial dilutions of EtBr. OD_600_ values were obtained by a plate reader at 18 h post-inoculation. **(C)** Eﬄux pump inhibitor CCCP sensitizes the AR-13^r^ mutant to EtBr. MT was grown in twofold serial dilutions of EtBr with or without a sub-inhibitory concentration of CCCP (4 nM). OD_600_ values were obtained by a plate reader at 18 h post-inoculation. Data were representative of 2–4 independent experiments each performed in triplicate; ^∗∗∗^*p* < 0.001; NS, not significant.

Comparative transcriptomic analysis revealed that the AR-13 resistance of this mutant is not due to changes in the RNA levels since no gene transcript was differentially expressed greater than 1.7-fold relative to the parental strain (data not shown). Thus, the observed stable AR-13 resistance in *Francisella* indicated an alteration in the inherent genomic information of the resistant mutant. Comparative genomic analysis was performed between the AR-13 resistant mutant and its parent wild type strain. This analysis identified three non-synonymous mutations in the AR-13 resistant mutant. Interestingly, two of them were found in genes encoding for outer membrane eﬄux proteins FTL_1107 (FtlC) and FTL_1865 (TolC) (Gil et al., 2006) with amino acid substitutions Leu236Pro and Glu441Lys, respectively (**Table 1**). TolC is the outer membrane channel component for multidrug eﬄux and type I secretion systems. The other mutation was in gene locus FTL_0600 (*wbtH* homolog in *F. novicida*) (Thomas et al., 2007) that is involved in O-antigen synthesis (amino acid substitution of Pro353Ser). Eﬄux pumps confer resistance to a variety of antibiotics and detergents in *Francisella* (Bina et al., 2008; Ahmad et al., 2010; Gestin et al., 2010). We hypothesized that the mutations in the eﬄux systems resulted in a gain of function mutation that increased eﬄux activity. As such, treatment with an eﬄux inhibitor would then sensitize the AR-13 resistant mutant to AR-13. To test our hypothesis, we utilized experimental approaches with AR-13 and the proton-mediated eﬄux pump inhibitor carbonyl cyanide *m*-chlorophenyl hydrazone (CCCP). Various concentrations of CCCP were tested against LVS to which dramatic reductions in growth were observed at >∼8 nM (Supplementary Figure 4). Thus, a 4 nM sub-inhibitory concentration of CCCP was chosen and was examined in combination with various concentrations of AR-13. We found that the AR-13 resistant mutant was more susceptible to AR-13 in the presence of sub-inhibitory concentrations of CCCP. The growth of the AR-13 resistant mutant was significantly decreased at 2.5 μg/ml AR-13 and nearly completely inhibited at 5 μg/ml AR-13 in the presence of CCCP while the strain grew normally at all concentrations of AR-13 (up to 10 μg/ml) in the absence of a sub-inhibitory concentration of the inhibitor (**Figure 5A**). In addition, the AR-13 resistant isolate also conferred decreased sensitivity to ethidium bromide (EtBr) which is mediated by TolC as previously observed (Gil et al., 2006) (**Figure 5B**), but does not affect sensitivity to kanamycin (Supplementary Figure 5). Similar to what was observed with AR-13, a sub-inhibitory concentration of CCCP sensitized the AR-13 resistant strain to EtBr (**Figure 5C**). In parallel, we examined independent mutants of two TolC orthologs, (FtlC; FTN_0779 in *F. novicida*) and FTL_1865 (TolC; FTN_1703 in *F. novicida*) as well as the O-antigen synthesis locus (FTL_0600; FTN_1421 in *F. novicida*), which were obtained from a BEI Resources transposon library (Gallagher et al., 2007), for susceptibility to AR-13. This showed that both *tolC* eﬄux genes, but not the O-antigen synthesis gene, confer intrinsic resistance to AR-13 in *F. novicida* (Supplementary Figure 6). These data provide strong evidence that eﬄux pumps mediate AR-13 resistance in *Francisella* and suggest that AR-13 has an intracellular target(s).

**Table 1:** Comparative genomic analysis of an AR-13 resistant (AR-13^r^) mutant and the wild-type (WT) identified three non-synonymous mutations.

Gene ID	Functions	cDNA pos/length	AA pos/length	WT	AR-13^r^	HGVS.p
*FTL_0600*	O-antigen synthesis	1057/1887	353/628	C	T	p.Pro353Ser
*FTL_1107*	Predicted outer membrane exflux protein	707/1374	236/457	T	C	p.Leu236Pro
*FTL_1865*	Outer membrane protein TolC precursor	1321/1530	441/509	C	T	p.Glu441Lys

### AR-13 Treatment of *F. tularensis* SchuS4 Infected Mice

**FIGURE 6:**
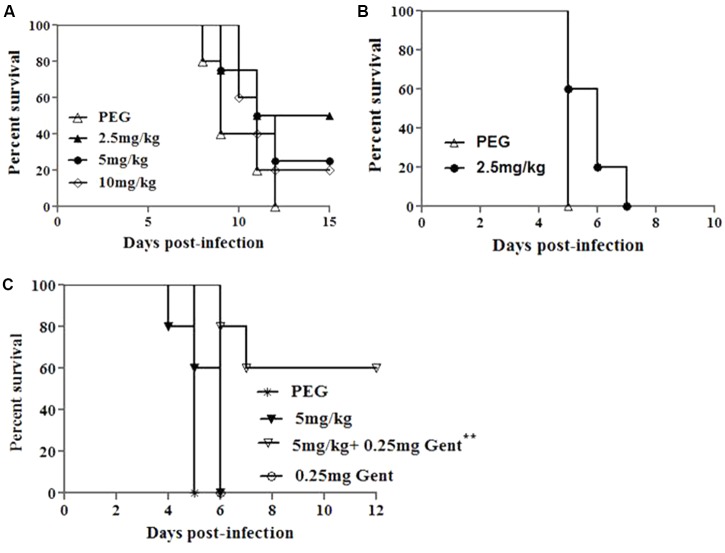
Protective effects of AR-13 in a mouse model of tularemia. **(A)** Survival curves of BALB/c mice infected by the I.N. route with 3 × 10^3^ CFU of LVS followed by AR-13 treatment. The infected mice (4 or 5 mice/group) were given 2.5, 5, or 10 mg of AR-13/kg/day, starting about 1 h post-infection, once daily from day 0 for 10 days. A control PEG group was included. **(B)** Survival curves of BALB/c mice (5 mice/group) infected by the I.N. route with 10 CFU of *F. tularensis* SchuS4 followed by AR-13 treatment. Infected mice were given 2.5 mg AR-13/kg/day starting about 1 h post-infection, once daily from day 0 until day 4. **(C)** Survival curves of BALB/c mice (5 mice/group) infected I.N. with 10 CFU of *F. tularensis* SchuS4 followed by AR-13 treatment. The infected mice were given 5 mg AR-13/kg/day, starting about 1 h post-infection, with or without 0.25 mg gentamicin/kg/day given I.P.. AR-13 was given once daily from day 0 until day 4; gentamicin was given once daily from day 0 until the end of the experiment. A control PEG group was included (^∗∗^ difference with respect to PEG and 0.25 mg gentamicin control groups, *p* < 0.01).

AR-13 exerts bactericidal effects on *Francisella* (**Figures 2B,D**) and causes minimal cytotoxicity to hMDMs (Supplementary Figure 3). Therefore, we tested the ability of AR-13 to protect mice from a lethal intranasal dose of *F. tularensis* SchuS4 or LVS. Toxicity of AR-13 in mice was first evaluated in non-infected mice I.P. treated with 10 mg AR-13/kg/day once daily for 10 consecutive days. These mice did not show any abnormal clinical signs (sickness, hair ruﬄing) which indicated that they can tolerate at least total 100 mg AR-13/kg. We next infected mice by the I.N. route with a lethal dose of LVS (3 × 10^3^ CFUs/mouse) and then treated the infected mice, starting about 1 h post-infection, once daily by I.P. injection with 2.5, 5, or 10 mg AR-13/kg/day in 200 μl PEG (Hoang et al., 2016) for 10 days. A PEG-only treated group was included as a control. As shown in **Figure 6A**, treatment of 2.5 mg AR-13/kg/day for 10 consecutive days provided the best protective effects (50% survival) from LVS infection (total drug of 25 mg AR-13/kg). There were no culturable bacteria in the lungs of these surviving mice at 15 days post-infection (data not shown). Because the 5 and 10 mg AR-13/kg/day treatments demonstrated less protective effects than mice given 2.5 mg AR-13/kg/day, but alone showed no toxicity at these doses, it is possible that the combination of LVS infection and these higher doses of AR-13 may be toxic to mice.

We then sought to evaluate the ability of AR-13 to control virulent Type A *F. tularensis* SchuS4 (a human clinical isolate) in the mouse infection model. Our previous publication showed that without treatment, mice died from days 4 to 6 following intranasal infection with 10 CFUs of *F. tularensis* SchuS4 (Hoang et al., 2016). Since AR-13 treatment (25 mg/kg total drug) only provided 50% protective effects (**Figure 6A**) the compound was also tested in combination with a sub-optimal dose of gentamicin, one of the primary antibiotics commonly used to treat *Francisella* infection (Hoang et al., 2016). Mice were infected with 10 CFUs of *F. tularensis* SchuS4/mouse via the I.N. route and then treated, starting about 1 h post-infection, with 2.5 mg (total dose 12.5 mg/kg) or 5 mg AR-13/kg/day (total dose 25 mg/kg), or with 5 mg AR-13/kg/day plus 0.25 mg gentamicin/kg/day (I.P.) once daily for 5 consecutive days. PEG and 0.25 mg gentamicin treated groups were included as controls. As shown in **Figures 6B,C**, AR-13 treatment prolonged survival of *F. tularensis* SchuS4 infected mice but did not protect mice from death. All PEG and gentamicin only treated mice died at day 5 or day 6 post-infection, respectively (**Figures 6B,C**). However, the combination of a sub-optimal dose of gentamicin with 5 mg AR-13/kg/day protected 60% of the infected mice from death (**Figure 6C**), with no CFU recovered from the lungs of surviving mice (data not shown). These data suggest that AR-13 could potentially be used as a combinational therapeutic to control *Francisella* infection.

## Discussion

There is a limited pool of antibiotics available for the treatment of tularemia in humans (Enderlin et al., 1994; Boisset et al., 2014). Although antibiotic treatment is frequently successful, relapse rates or complete failure of treatment up to 33% has been reported (Enderlin et al., 1994). Resistance to conventional antibiotics in *Francisella* has not been clinically described (Urich and Petersen, 2008); however, antibiotic resistant strains could be malevolently created instituting a significant threat as a biological weapon. Thus, it has become paramount to develop new antimicrobial strategies to control this potentially dangerous microbial pathogen (Boisset et al., 2014). Here we show that AR-13, a derivative of a cyclooxygenase-2 inhibitor, exhibits direct killing activity against different *Francisella* subspecies including *F. tularensis* SchuS4, LVS (**Figure 2**), and *F. novicida* (data not shown). *M. tuberculosis and S. aureus* have been demonstrated to be susceptible to direct killing by AR-13 (Salunke et al., 2015). While *P. aeruginosa* and *Salmonella* serovars tested in our laboratory were not susceptible to the drug, *L. monocytogenes* and methicillin-resistant *S. aureus* showed strong susceptibility to direct killing (Supplementary Figure 2).

The nature of the antimicrobial activity [defined as killing 99.9% of a bacterial inoculum within a 24 h exposure period (Pankey and Sabath, 2004)] of AR-13 showed that it exerted a time-kill kinetic effect on LVS (approximately 2- and 4-log CFU reduction at 8 h post-treatment for *F. tularensis.* SchuS4 and LVS, respectively) (**Figures 2B,D**). These findings suggest that the antibacterial effect of AR-13 is likely not mediated by rapid disruption of membrane integrity, but rather that the target of the compound is intracellular. Unlike the bactericidal effects of AR-13, AR-16, another derivative of cyclooxygenase-2 inhibitor, exerted bacteriostatic anti-*Francisella* activity (**Figure 2C**). While bacteriostatic agents have a place in the toolkit of compounds to treat bacterial infections, our work here focused on the bactericidal activity of AR-13.

The inhibitory effect of AR-13 on LVS in hMDMs, a primary cellular target of *Francisella* spp., was moderate (**Figure 3**). This was surprising, as other derivatives of the parental compound, such as AR-12, penetrate cells and kill intracellularly by the induction of autophagy (Chiu et al., 2009b; Hoang et al., 2014). The limited activity of AR-13 against intracellular *Francisella* suggests that the molecule may enter macrophages poorly, lack co-localization with the bacteria, or be intracellularly degraded. Future work will address this issue and if necessary, AR-13 can be derivatized or encapsulated to enhance intracellular targeting.

To identify the potential target of the antibacterial activity of AR-13, we sought to create a resistant mutant. By repeated passage in increasing drug concentrations, we were able to select for a stable resistant mutant to AR-13 (**Figure 4**). Comparative genomic sequencing combined with biological assays demonstrated that this resistance is likely mediated by multidrug eﬄux pumps involving genes FTL_1107 and FTL_1865. Amino acid mutations at Leu236Pro and Glu441Lys, respectively, were identified in these eﬄux pumps which presumably increase general or AR-13 specific eﬄux activity. RNAseq data showed no increase in eﬄux pump gene transcription in these mutants, further suggesting increased eﬄux as the mechanism behind the increase in AR-13 resistance. To the best of our knowledge, no information exists about these mutations regarding enhanced activity of eﬄux pumps in Gram-negative bacteria. The role of eﬄux in resistance was confirmed with the use of an eﬄux pump inhibitor or inactivation of the eﬄux pump, both abolishing the observed resistance (**Figures 5A–C** and Supplementary Figure 6). Interestingly, the AR-13 resistant mutant also conferred resistance to ethidium bromide (**Figures 5B,C**), which is known to be mediated, in part, by the eﬄux pump TolC (Gil et al., 2006). Investigation of these mutations in *Francisella* will shed the light on the roles of these amino acid residues on the activities of eﬄux pumps for both antimicrobial and detergent resistance. While our selection screen failed to identify the AR-13 target, these data together with the result from time-kill kinetics, suggest an intracellular target for AR-13. From a translational perspective, continued work to identify AR-13 resistance mechanisms will help guide the development of AR-13 derivatives with more potent activity as well as new therapeutic strategies to combat *Francisella* infections.

Using *Francisella* infected mice, it was demonstrated that AR-13 possessed promising *in vivo* anti-*Francisella* activity. Despite infection with a lethal dose of LVS, two of four mice treated with 2.5 mg/kg/day (total 25 mg/kg for the whole course of treatment) of AR-13 recovered and were healthy at the study endpoint, while none of the vehicle-treated control mice survived. It is noteworthy that higher doses of AR-13 (5 mg/kg and 10 mg/kg/day) resulted in less protective effects, suggesting potential toxicity of the drug at these doses in infected animals (**Figure 6A**). Since the most protective dose of AR-13 for LVS-infected mice was a total of 25 mg/kg, we sought to examine whether this dose could protect mice from a lethal dose of the human pathogen, *F. tularensis* SchuS4. Treatment with this dose prolonged survival of the infected mice but did not protect the mice from death (**Figure 6B**). However, when combined with the sub-optimal dose of gentamicin, three of five mice were protected while all infected mice treated with the sub-optimal dose of gentamicin died (**Figure 6C**). *F. tularensis* is primarily an intracellular pathogen of macrophages, and the compound was shown to have limited activity against *Francisella* in macrophages. This may partially explain why better protection was not observed in mice. However, gentamicin, an antibiotic that has poor cellular penetrating activity, augmented AR-13 activity, likely by enhancing the permeability of the eukaryotic and/or bacterial envelope to AR-13 (the latter during *Francisella’s* extracellular phase). However, these data provide strong evidence that AR-13, either with or without traditional antibiotic augmentation, is a promising lead candidate as a drug to aid control of tularemia in humans.

## Author Contributions

All authors listed have made a substantial, direct and intellectual contribution to the work, and approved it for publication. KH and JG conceived and designed the study. KH, HA, JF, DG, PW, and HC performed the experiments and analyzed the data. KH, LS, and JG: wrote and revised the manuscript.

## Conflict of Interest Statement

The authors declare that the research was conducted in the absence of any commercial or financial relationships that could be construed as a potential conflict of interest.

## Footnotes

1 https://www.beiresources.org

2 http://genomebiology.biomedcentral.com/articles/10.1186/s13059-014-0577-x

3 http://bio-bwa.sourceforge.net

4 https://www.ncbi.nlm.nih.gov/genome/511?genome\_assembly\_id=167333

5 https://software.broadinstitute.org/gatk/documentation/tooldocs/current/org\ _broadinstitute\_gatk\_tools\_walkers\_haplotypecaller_HaplotypeCaller.php

6 http://snpeff.sourceforge.net

7 http://www.ncbi.nlm.nih.gov/genome/511?genome_assembly_id=167333

8 http://bio-bwa.sourceforge.net/

9 http://www-huber.embl.de/users/anders/HTSeq/doc/count.html

10 http://genomebiology.com/2014/15/12/550

11 https://www.ncbi.nlm.nih.gov/geo/

12 https://www.ncbi.nlm.nih.gov/sra

## References

[B1] Ahmad S. Hunter L. Qin A. P. Mann B. J. van Hoek M. L. (2010). Azithromycin effectiveness against intracellular infections of *Francisella*. *BMC Microbiol.* 10:123 10.1186/1471-2180-10-123 PMC2881020 20416090

[B2] Baxter B. K. DiDone L. Ogu D. Schor S. Krysan D. J. (2011). Identification, *in vitro* activity and mode of action of phosphoinositide-dependent-1 kinase inhibitors as antifungal molecules. *ACS Chem. Biol.* 6 502–510. 10.1021/cb100399x 21294551 PMC3098953

[B3] Bina X. R. Lavine C. L. Miller M. A. Bina J. E. (2008). The AcrAB RND eﬄux system from the live vaccine strain of *Francisella tularensis* is a multiple drug eﬄux system that is required for virulence in mice. *FEMS Microbiol. Lett.* 279 226–233. 10.1111/j.1574-6968.2007.01033.x 18179581

[B4] Boisset S. Caspar Y. Sutera V. Maurin M. (2014). New therapeutic approaches for treatment of tularaemia: a review. *Front. Cell. Infect. Microbiol.* 4:40 10.3389/fcimb.2014.00040 PMC3975101 24734221

[B5] Chiu H. C. Kulp S. K. Soni S. Wang D. Gunn J. S. Schlesinger L. S. (2009a). Eradication of intracellular *Salmonella enterica* serovar Typhimurium with a small-molecule, host cell-directed agent. *Antimicrob. Agents Chemother.* 53 5236–5244. 10.1128/AAC.00555-09 19805568 PMC2786354

[B6] Chiu H. C. Lee S. L. Kapuriya N. Wang D. Chen Y. R. Yu S. L. (2012). Development of novel antibacterial agents against methicillin-resistant *Staphylococcus aureus*. *Bioorg. Med. Chem.* 20 4653–4660. 10.1016/j.bmc.2012.06.018 22750009 PMC3401364

[B7] Chiu H. C. Soni S. Kulp S. K. Curry H. Wang D. Gunn J. S. (2009b). Eradication of intracellular *Francisella tularensis* in THP-1 human macrophages with a novel autophagy inducing agent. *J. Biomed. Sci.* 16:110 10.1186/1423-0127-16-110 PMC2801672 20003180

[B8] Chiu H. C. Yang J. Soni S. Kulp S. K. Gunn J. S. Schlesinger L. S. (2009c). Pharmacological exploitation of an off-target antibacterial effect of the cyclooxygenase-2 inhibitor celecoxib against *Francisella tularensis*. *Antimicrob. Agents Chemother.* 53 2998–3002. 10.1128/Aac.00048-09 19398640 PMC2704645

[B9] Clay C. D. Soni S. Gunn J. S. Schlesinger L. S. (2008). Evasion of complement-mediated lysis and complement C3 deposition are regulated by *Francisella tularensis* lipopolysaccharide O antigen. *J. Immunol.* 181 5568–5578. 10.4049/jimmunol.181.8.5568 18832715 PMC2782685

[B10] Dai S. Mohapatra N. P. Schlesinger L. S. Gunn J. S. (2012). The acid phosphatase AcpA is secreted in vitro and in macrophages by *Francisella* spp. *Infect. Immun.* 80 1088–1097. 10.1128/IAI.06245-11 22184418 PMC3294640

[B11] Enderlin G. Morales L. Jacobs R. F. Cross J. T. (1994). Streptomycin and alternative agents for the treatment of tularemia: review of the literature. *Clin. Infect. Dis.* 19 42–47. 7948556 10.1093/clinids/19.1.42

[B12] Gallagher L. A. Ramage E. Jacobs M. A. Kaul R. Brittnacher M. Manoil C. (2007). A comprehensive transposon mutant library of *Francisella novicida*, a bioweapon surrogate. *Proc. Natl. Acad. Sci. U.S.A.* 104 1009–1014. 10.1073/pnas.0606713104 17215359 PMC1783355

[B13] Gestin B. Valade E. Thibault F. Schneider D. Maurin M. (2010). Phenotypic and genetic characterization of macrolide resistance in *Francisella tularensis* subsp. *holarctica* biovar I. *J. Antimicrob. Chemother.* 65 2359–2367. 10.1093/jac/dkq315 20837574

[B14] Gil H. Platz G. J. Forestal C. A. Monfett M. Bakshi C. S. Sellati T. J. (2006). Deletion of TolC orthologs in *Francisella tularensis* identifies roles in multidrug resistance and virulence. *Proc. Natl. Acad. Sci. U.S.A.* 103 12897–12902. 10.1073/pnas.0602582103 16908853 PMC1568944

[B15] Gurcan S. (2014). Epidemiology of tularemia. *Balkan Med. J.* 31 3–10. 10.5152/balkanmedj.2014.13117 25207161 PMC4115998

[B16] Hestvik G. Warns-Petit E. Smith L. A. Fox N. J. Uhlhorn H. Artois M. (2015). The status of tularemia in Europe in a one-health context: a review. *Epidemiol. Infect.* 143 2137–2160. 10.1017/S0950268814002398 25266682 PMC9506979

[B17] Hoang K. Wang Y. Lin J. (2012). Identification of genetic loci that contribute to *Campylobacter* resistance to fowlicidin-1, a chicken host defense peptide. *Front. Cell. Infect. Microbiol.* 2:32 10.3389/Fcimb.2012.00032 PMC3417529 22919624

[B18] Hoang K. V. Borteh H. M. Rajaram M. V. S. Peine K. J. Curry H. Collier M. A. (2014). Acetalated dextran encapsulated AR-12 as a host-directed therapy to control *Salmonella* infection. *Int. J. Pharm.* 477 334–343. 10.1016/j.ijpharm.2014.10.022 25447826 PMC4267924

[B19] Hoang K. V. Curry H. Collier M. A. Borteh H. Bachelder E. Schlesinger L. S. (2016). Needle-free delivery of acetalated dextran-encapsulated AR-12 protects mice from *Francisella tularensis* lethal challenge. *Antimicrob. Agents Chemother.* 60 2052–2062. 10.1128/AAC.02228-15 26787696 PMC4808193

[B20] Hoang K. V. Stern N. J. Saxton A. M. Xu F. Zeng X. Lin J. (2011). Prevalence, development, and molecular mechanisms of bacteriocin resistance in *Campylobacter*. *Appl. Environ. Microbiol.* 77 2309–2316. 10.1128/AEM.02094-10 21278269 PMC3067428

[B21] Jones C. J. Newsom D. Kelly B. Irie Y. Jennings L. K. Xu B. (2014). ChIP-Seq and RNA-Seq reveal an AmrZ-mediated mechanism for cyclic di-GMP synthesis and biofilm development by *Pseudomonas aeruginosa*. *PLOS Pathog.* 10:e1003984 10.1371/journal.ppat.1003984 PMC3946381 24603766

[B22] Jones C. L. Napier B. A. Sampson T. R. Llewellyn A. C. Schroeder M. R. Weiss D. S. (2012). Subversion of host recognition and defense systems by *Francisella* spp. *Microbiol. Mol. Biol. Rev.* 76 383–404. 10.1128/MMBR.05027-11 22688817 PMC3372254

[B23] Kelly B. J. Fitch J. R. Hu Y. Corsmeier D. J. Zhong H. Wetzel A. N. (2015). Churchill: an ultra-fast, deterministic, highly scalable and balanced parallelization strategy for the discovery of human genetic variation in clinical and population-scale genomics. *Genome Biol.* 16 6 10.1186/s13059-014-0577-x PMC4333267 25600152

[B24] Kingry L. C. Petersen J. M. (2014). Comparative review of *Francisella tularensis* and *Francisella novicida*. *Front. Cell Infect. Microbiol.* 4:35 10.3389/fcimb.2014.00035 PMC3952080 24660164

[B25] Kosker M. Sener D. Kilic O. Akil F. Yilmaz M. Ozturk O. (2013). A case of oculoglandular tularemia resistant to medical treatment. *Scand. J. Infect. Dis.* 45 725–727. 10.3109/00365548.2013.796089 23746340

[B26] Kugeler K. J. Mead P. S. Janusz A. M. Staples J. E. Kubota K. A. Chalcraft L. G. (2009). Molecular epidemiology of *Francisella tularensis* in the United States. *Clin. Infect. Dis.* 48 863–870. 10.1086/597261 19245342

[B27] Maurin M. (2014). New anti-infective strategies for treatment of tularemia. *Front. Cell. Infect. Microbiol.* 4:115 10.3389/fcimb.2014.00115 PMC4137221 25191647

[B28] Origgi F. C. Frey J. Pilo P. (2014). Characterisation of a new group of *Francisella tularensis* subsp. *holarctica* in Switzerland with altered antimicrobial susceptibilities, 1996 to 2013. *Euro Surveill.* 19:20858 10.2807/1560-7917.ES2014.19.29.20858 25080140

[B29] Oyston P. C. (2008). *Francisella tularensis*: unravelling the secrets of an intracellular pathogen. *J. Med. Microbiol.* 57(Pt 8), 921–930. 10.1099/jmm.0.2008/000653-0 18628490

[B30] Oyston P. C. Sjostedt A. Titball R. W. (2004). Tularaemia: bioterrorism defence renews interest in *Francisella tularensis*. *Nat. Rev. Microbiol.* 2 967–978. 10.1038/nrmicro1045 15550942

[B31] Pammit M. A. Budhavarapu V. N. Raulie E. K. Klose K. E. Teale J. M. Arulanandam B. P. (2004). Intranasal interleukin-12 treatment promotes antimicrobial clearance and survival in pulmonary *Francisella tularensis* subsp. *novicida* infection. *Antimicrob. Agents Chemother.* 48 4513–4519. 10.1128/AAC.48.12.4513-4519.2004 15561819 PMC529201

[B32] Pankey G. A. Sabath L. D. (2004). Clinical relevance of bacteriostatic versus bactericidal mechanisms of action in the treatment of Gram-positive bacterial infections. *Clin. Infect. Dis.* 38 864–870. 10.1086/381972 14999632

[B33] Perez-Castrillon J. L. Bachiller-Luque P. Martin-Luquero M. Mena-Martin F. J. Herreros V. (2001). Tularemia epidemic in northwestern Spain: clinical description and therapeutic response. *Clin. Infect. Dis.* 33 573–576. 10.1086/322601 11462198

[B34] Salunke S. B. Azad A. K. Kapuriya N. P. Balada-Llasat J. M. Pancholi P. Schlesinger L. S. (2015). Design and synthesis of novel anti-tuberculosis agents from the celecoxib pharmacophore. *Bioorg. Med. Chem.* 23 1935–1943. 10.1016/j.bmc.2015.03.041 25818768

[B35] Schlesinger L. S. Bellingerkawahara C. G. Payne N. R. Horwitz M. A. (1990). Phagocytosis of *Mycobacterium tuberculosis* is mediated by human monocyte complement receptors and complement component C3. *J. Immunol.* 144 2771–2780. 2108212

[B36] Staples J. E. Kubota K. A. Chalcraft L. G. Mead P. S. Petersen J. M. (2006). Epidemiologic and molecular analysis of human tularemia, United States, 1964-2004. *Emerg. Infect. Dis.* 12 1113–1118. 10.3201/eid1207.051504 16836829 PMC3291054

[B37] Sutera V. Levert M. Burmeister W. P. Schneider D. Maurin M. (2014). Evolution toward high-level fluoroquinolone resistance in *Francisella* species. *J. Antimicrob. Chemother.* 69 101–110. 10.1093/jac/dkt321 23963236

[B38] Thomas L. D. Schaffner W. (2010). Tularemia pneumonia. *Infect. Dis. Clin. North Am.* 24 43–55. 10.1016/j.idc.2009.10.012 20171544

[B39] Thomas R. M. Titball R. W. Oyston P. C. F. Griffin K. Waters E. Hitchen P. G. (2007). The immunologically distinct O antigens from *Francisella tularensis* subspecies *tularensis* and *Francisella novicida* are both virulence determinants and protective antigens. *Infect. Immun.* 75 371–378. 10.1128/Iai.01241-06 17074846 PMC1828428

[B40] Urich S. K. Petersen J. M. (2008). In vitro susceptibility of isolates of *Francisella tularensis* types A and B from North America. *Antimicrob. Agents Chemother.* 52 2276–2278. 10.1128/AAC.01584-07 18411318 PMC2415761

