# Supplementary material for: AR-13, a Celecoxib Derivative, Directly Kills Francisella In Vitro and Aids Clearance and Mouse Survival In Vivo
Source: Front Microbiol. 2017 Sep 11;8:1695. doi: 10.3389/fmicb.2017.01695 (PMC5600997; doi:10.3389/fmicb.2017.01695)
Supplement: Supplementary file 1 [file Image_1.PDF]

Supplementary Material

AR-13, a celecoxib derivative, directly kills *Francisella in vitro* and aids clearance and mouse survival *in vivo*

Ky Van Hoang[1], Haley E. Adcox[1], James R. Fitch[2], David M. Gordon[2], Heather Curry[1], Larry S. Schlesinger[1], Peter White[2,3] and John S. Gunn[1]*

[1]Center for Microbial Interface Biology, Department of Microbial Infection and Immunity, The Ohio State University, Columbus OH 43210; [2]The Institute for Genomic Medicine, Nationwide Children's Hospital, 700 Children's Drive, Columbus OH 43205; [3]The Ohio State University College of Medicine, Department of Pediatrics, Columbus OH 43210.

Corresponding author: John S. Gunn, E-mail: gunn.43@osu.edu

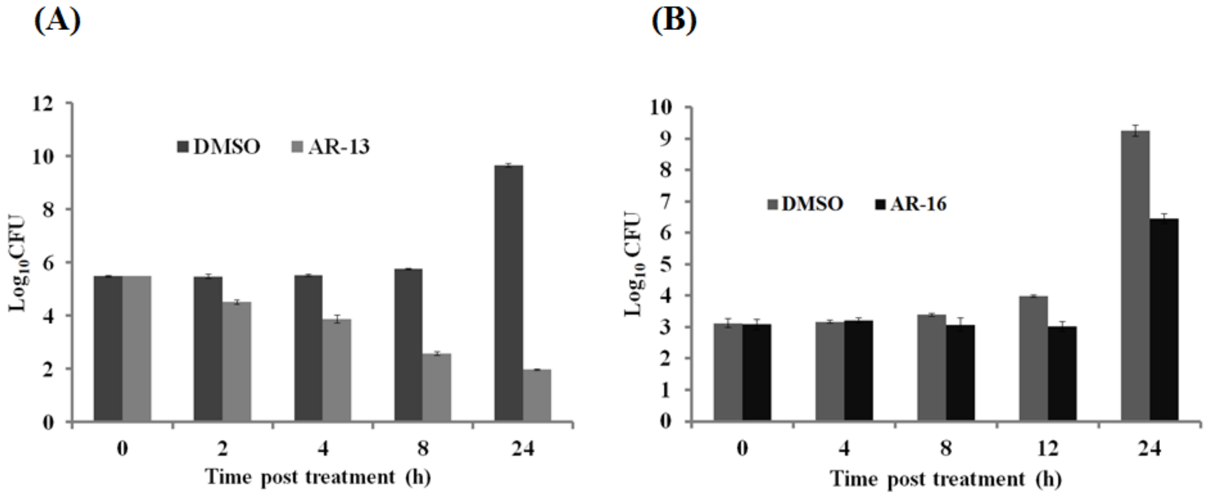

Supplementary Figure 1. Approximately 5-6x10^5 CFUs of LVS were incubated while shaking at 37°C in 1 ml mTSB containing 10 µg of AR-13 (A) or AR-16 (B). Viable bacteria were evaluated at different time-points by serial dilution and plating. Data are representative of three independent experiments, each performed in triplicate.

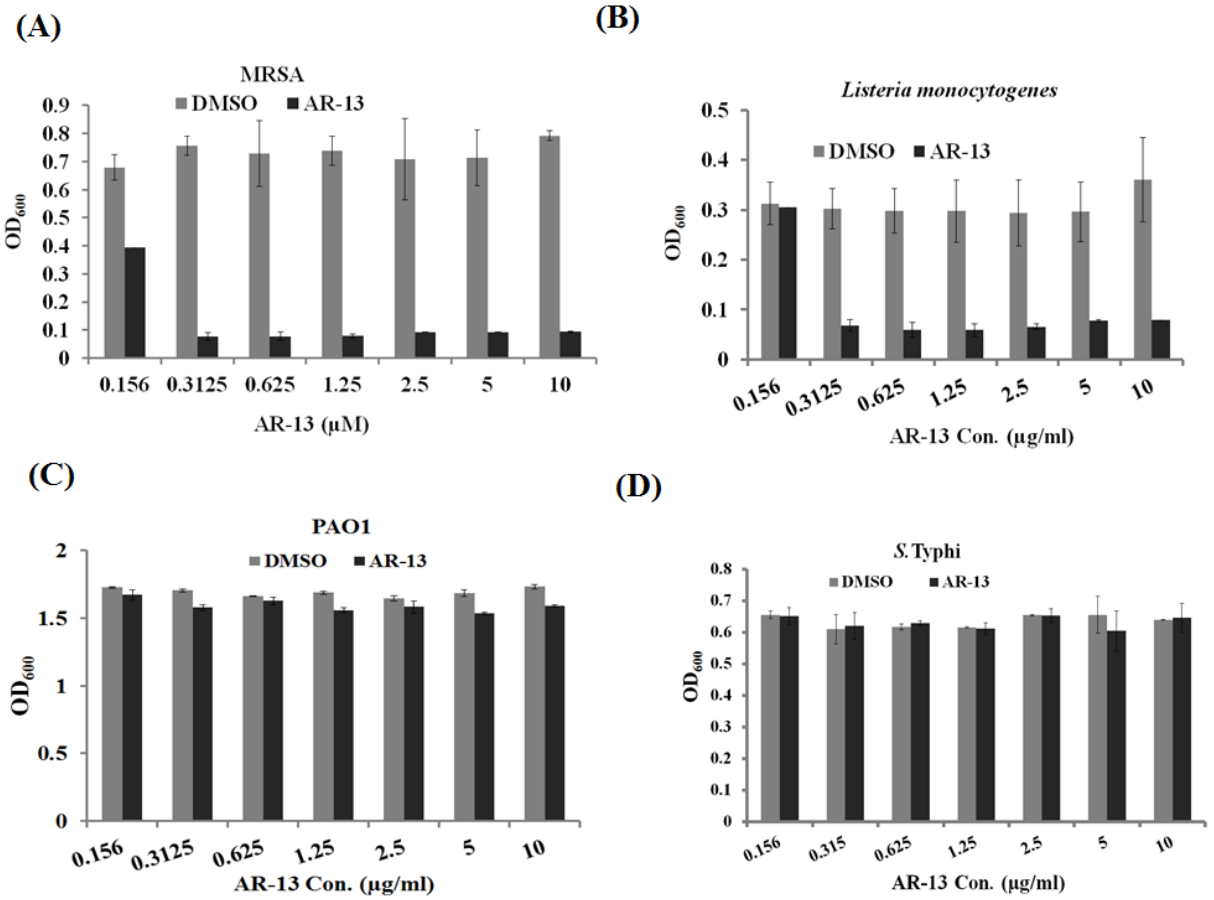

Supplementary Figure 2. Bacteria were grown in 2-fold serial dilutions of AR-13 in (A) mTSB (MRSA, strain JE2), (B) BHI (*L. monocytogenes,* strain 10403S), (C) LB (*P. aeruginosa* strain PAO1) and (D) *S.* Typhi Ty2. Optical densities at 600nm (OD_{600}) were measured by a plate reader at 24h post-inoculation. Two to three independent experiments were performed for each bacterium, with triplicate cultures in each experiment. Representative data are presented.

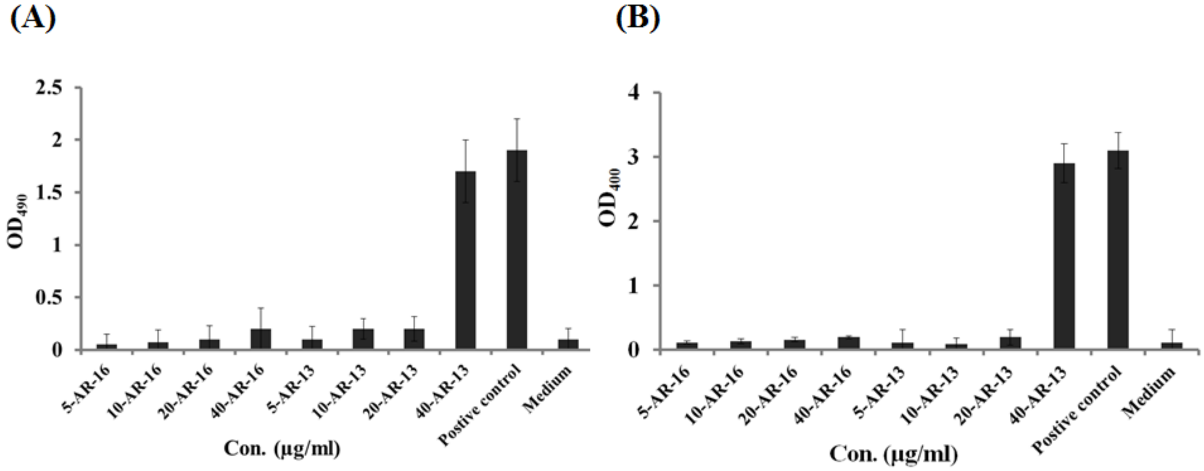

Supplementary Figure 3. Cytotoxicity of human monocyte-derived macrophages (hMDMs) cultured in the absence or presence of AR-13 or AR-16 for 24h (A) and 48h (B). Cytotoxicity was determined as LDH release measured at OD_{490}. A positive control (0.2% Triton X-100) was included. Experiments were repeated two times (each in triplicate) and the data from one experiment is presented.

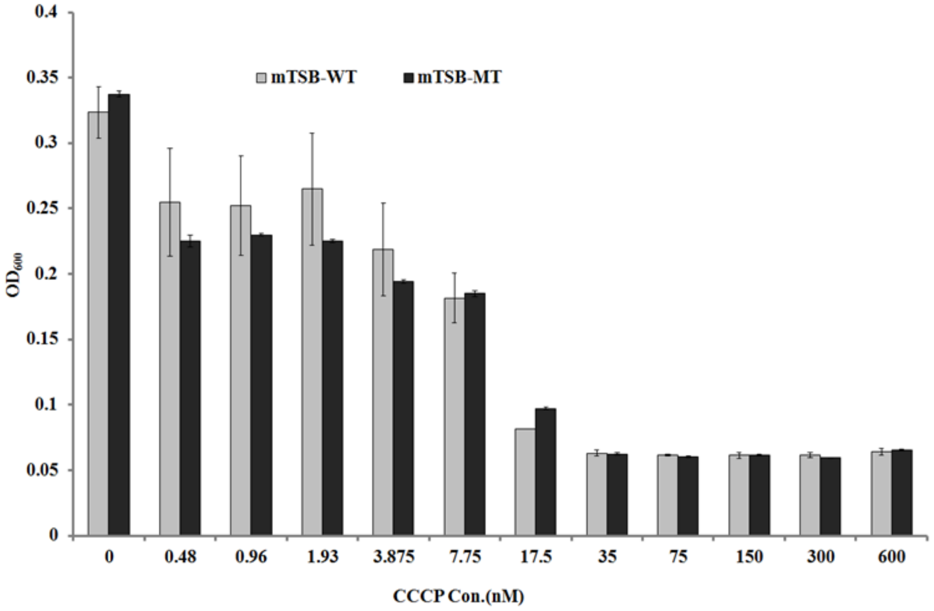

Supplementary Figure 4. Growth curve of a AR-13^r mutant (MT) and wild type LVS (WT) in varied concentrations of CCCP. LVS was grown in a 2-fold serial dilution of CCCP in mTSB. Optical densities at 600nm (OD_{600}) were measured by a plate reader at 18h post-inoculation. Experiments were repeated two times (each in triplicate) and the data from one experiment was presented.

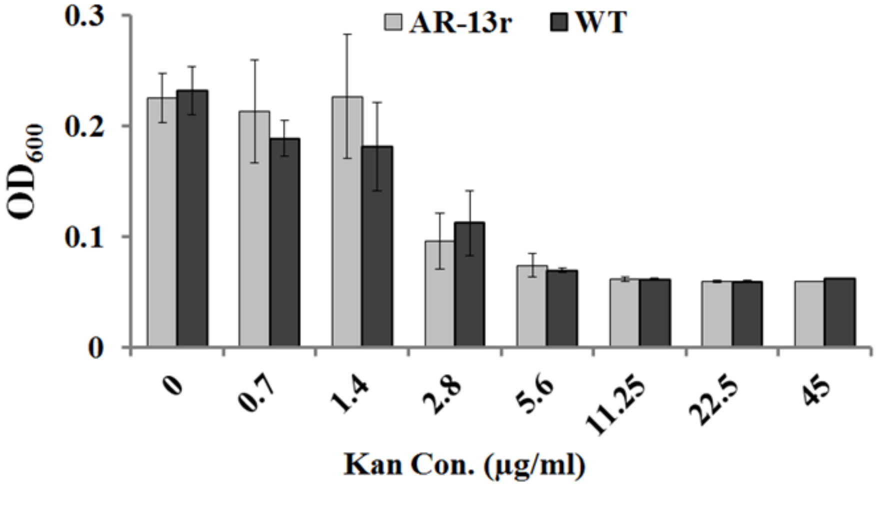

Supplementary Figure 5. An AR-13^r mutant does not confer resistance to kanamycin. LVS was grown in 2-fold serial dilutions of kanamycin in mTSB. Optical densities at 600nm (OD_{600}) were

measured by a plate reader at 18h post-inoculation. Experiments were repeated two times (each in triplicate) and the data from one experiment was presented.

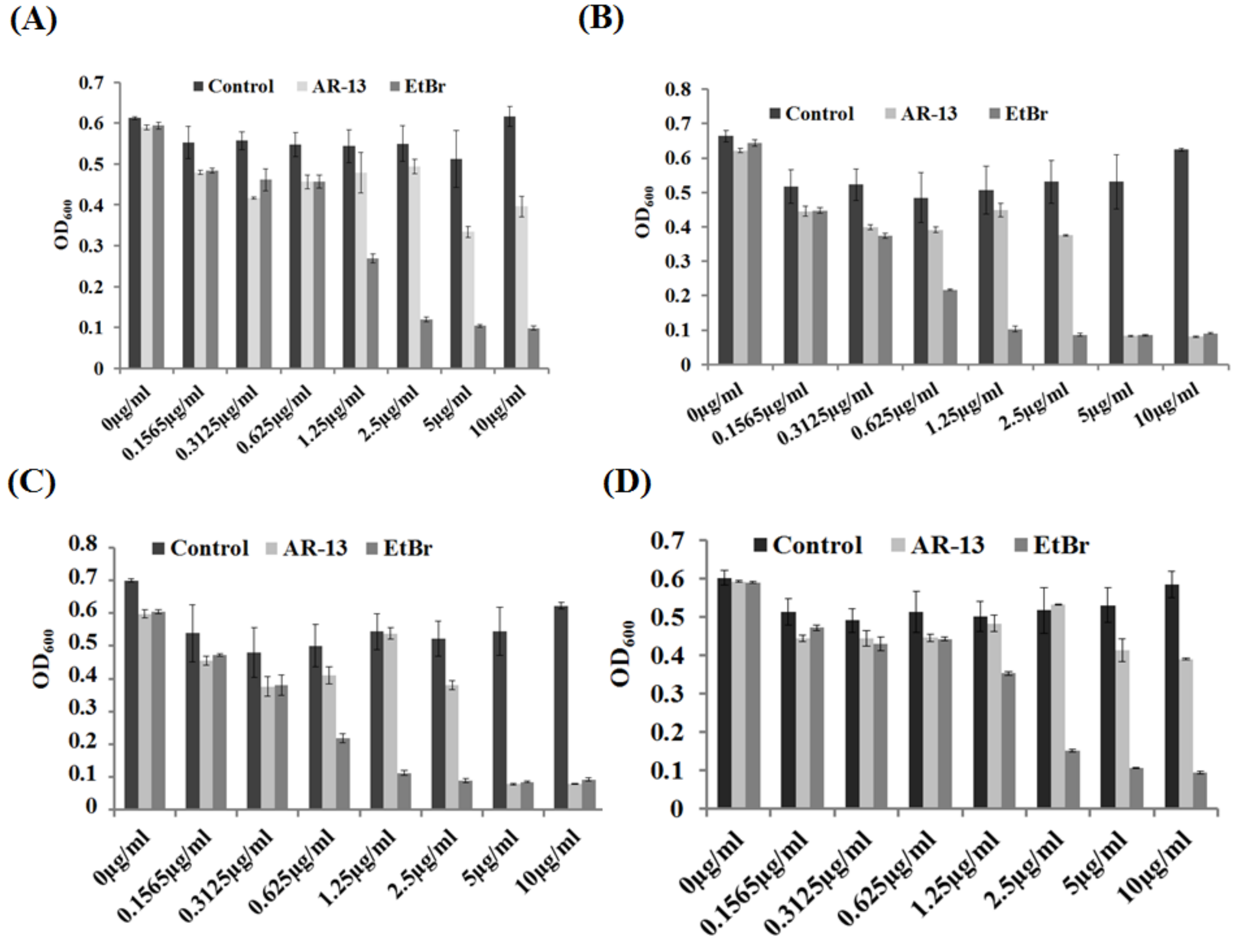

Supplementary Figure 6. Mutations in efflux pump genes but not an O-antigen synthesis gene (*wbtH*) confer AR-13 resistance in *F. novicida (Fn)*. Wild type *Fn*, together with efflux pump and *wbtH* mutants, were examined for sensitivity to AR-13 in mTSB; ethidium bromide was included as a control. (A) The growth of wild type *Fn* was partially inhibited by >5 µg of AR-13/ml and >1.25 µg of EtBr. Transposon insertions in (B) *tolC* (FTN_0779) and (C) *ftlC* (FTN_1703) increased susceptibility of these mutants to AR-13 (no growth was observed at 5 µg of AR-13/ml). (D) A *wbtH* (FTN_1421) mutant is only weakly affected by AR-13 concentrations of >5 µg of AR-13/ml. The experiment was performed 2-3 times in triplicate with at least three independent transposon mutants obtained from a BEI Resources library for each gene.
